# Modified BEST-J Score Model Predicts Bleeding after Endoscopic Submucosal Dissection with Fewer Factors

**DOI:** 10.3390/cancers14225555

**Published:** 2022-11-12

**Authors:** Tomoyuki Okada, Tsuyoshi Mikamo, Wataru Hamamoto, Taku Iwamoto, Toshiaki Okamoto, Kazunori Maeda, Atsushi Yanagitani, Kiwamu Tanaka, Hajime Isomoto, Naoyuki Yamaguchi

**Affiliations:** 1 Tottori Prefectural Central Hospital, Tottori 680-0901, Japan; 2 Division of Medicine and Clinical Science, Faculty of Medicine, Tottori University, Yonago 683-8504, Japan; 3 Department of Endoscopy, Nagasaki University Hospital, Nagasaki 852-8501, Japan

**Keywords:** early gastric cancer, gastric endoscopic submucosal dissection, postoperative bleeding, BEST-J score, prognostic nutritional index

## Abstract

**Simple Summary:**

BEST-J score is a model for predicting bleeding after gastric endoscopic submucosal dissection (ESD); however, it is complicated. Several post-ESD bleeding prediction models exist but have not been externally validated. Moreover, there are no post-ESD bleeding prediction models that incorporate nutritional indicators. This study aimed to predict post-ESD bleeding more simply by incorporating nutritional indicators and verify generalizability using an external cohort. This study could more quickly predict post-ESD bleeding.

**Abstract:**

This study constructed a simplified post-endoscopic submucosal dissection (ESD) prediction model with a prognostic nutritional index (PNI). A total of 449 patients who underwent gastric ESD was included, divided with a ratio of 2:1, and assigned to the model or validation cohort. A prediction model of post-ESD (modified BEST-J score) was constructed using the model cohort. The modified BEST-J score was evaluated by comparing its accuracy to the BEST-J score in the validation cohort. Within 4 weeks of ESD, melena, hematemesis, or a 2 g/dL or greater decrease in hemoglobin level that required esophagogastroduodenoscopy was defined as post-ESD bleeding. In the model cohort, 299 patients were enrolled and 25 (8.4%) had post-ESD bleeding. Independent risk factors for post-ESD bleeding were use of P2Y12RA, tumor size > 30 mm, location of lesion at lower one-third of the stomach, and PNI ≤ 47.9. Constructing the modified BEST-J score based on these variables, the sensitivity, specificity, and positive likelihood ratio were 73.9%, 78.1%, and 3.37. When comparing the modified BEST-J score to the BEST-J score in the validation cohort, no significant difference was observed by ROC-AUC (0.77 vs. 0.75, *p* = 0.81). Modified BEST-J score can predict post-ESD bleeding more simply, with the same accuracy as the BEST-J score.

## 1. Introduction

Gastric endoscopic submucosal dissection (ESD) is a useful and minimally invasive treatment. ESD has a similar 5-year overall survival, disease-specific survival, and a lower risk of complications from surgery [1,2]. Moreover, ESD is minimally invasive and conserves the stomach, improving quality of life [2]. ESD-related complications include perforation and delayed bleeding, which can have severe outcomes, requiring blood transfusions and emergency endoscopic hemostasis [3,4]. Several studies report that age, comorbidity, use of antithrombotics or anticoagulants, time taken for ESD, tumor location, and tumor size are related to delayed bleeding [3,4,5,6]. Hatta et al. reported a model that predicts post-ESD bleeding: BEST-J score [7]. The BEST-J score was constructed based on a large-scale multicenter study in Japan and demonstrated good discrimination and calibration [7]. However, the BEST-J score required a combination of many variables, which was complicated. Regarding postoperative complications, preoperative nutritional states affect postoperative outcomes and some nutritional indicators have been reported [8,9,10,11,12]. The prognostic nutritional index (PNI) is a nutritional indicator calculated by total lymphocyte count and serum albumin level, suggested by Onodera et al. [13]. Several studies have shown that preoperative PNI states for gastrointestinal cancer are useful predictors of postoperative complications [14,15,16]. To our knowledge, a few models that predict post-ESD bleeding exist [7,17]. Moreover, there is no predictive model of post-ESD bleeding, considering PNI, including the BEST-J score. Hence, this study sought to develop a simplified post-ESD prediction model that incorporates the PNI based on the BEST-J score. 

## 2. Materials and Methods

### 2.1. Study Design

This single-center retrospective observational study was approved by the Institutional Review Board of Prefectural Central Hospital (Tottori, Japan; approval number: 2020-86) and performed according to the Declaration of Helsinki [18]. Informed consent was obtained from the hospital website by the opt-out method. 

**Figure 1:**
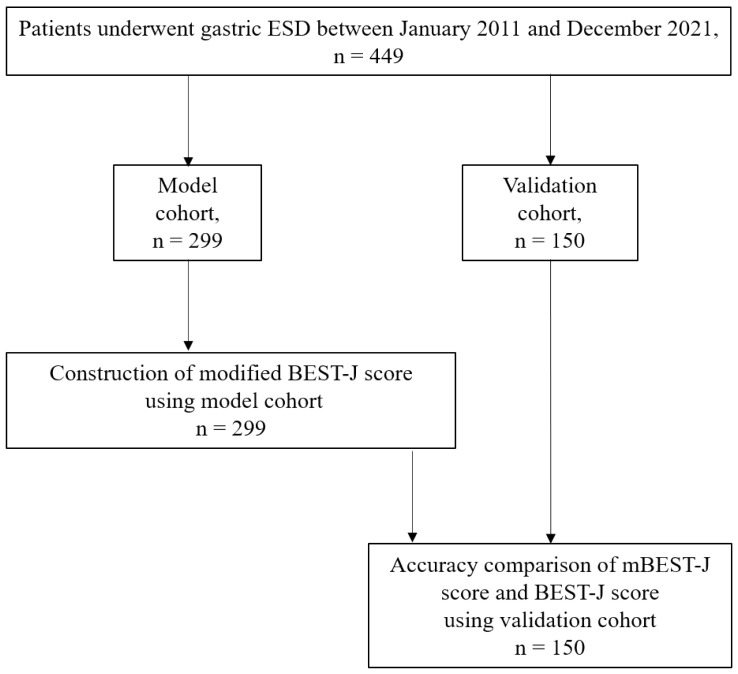
Study design flowchart.

Three steps were performed in this study. First, using the model cohort, components in the post-ESD bleeding model were identified using the BEST-J score and PNI. Regarding validity, the constructed predictive model was checked in the model cohort. Finally, using another validation cohort, we evaluated the validity of the predictive model by comparing it to the BEST-J score. Cases undergoing gastric ESD from January 2011 to December 2021 were randomly divided in a 2:1 ratio and assigned to either the model or validation cohort (Figure 1).

Cases were assigned to prevent overlap between the two cohorts and cases undergoing multiple ESDs were assigned to one cohort. All data were collected from an inclusive, computerized hospital database. 

### 2.2. Patients 

Patients treated with gastric ESD at the Tottori Prefectural Central Hospital (Tottori, Japan) from January 2011 to December 2021 participated in this study. Patients who discontinued treatment in the middle of ESD were excluded. Patients treated with laparoscopic and endoscopic cooperative surgery and those who refused to participate in this study were also excluded. 

### 2.3. ESD Procedure 

ESD was performed on lesions diagnosed or strongly suspected of early gastric cancer according to the guidelines for ESD produced by the Japan Gastroenterological Endoscopy Society (JGES) [19]. Based on previous reports [20,21,22,23], ESD was performed in a consistent procedure. First, the scope of the resected lesion was marked with a high-frequency generator (ERBR, Elektromed-VIO300D; Tubingen, Germany). Then, a mixture of glycerin solution, sodium hyaluronate, and indigo carmine was injected into the submucosa to lift the lesion. After swelling of the submucosa, the submucosal layer of the lesion was dissected with a diathermy knife (FlushKnife N-S, DK2620JI; Fujifilm, Tokyo, Japan or dual knife, KD-655L; Olympus, Tokyo, Japan) or an insulation-tipped diathermy knife (IT-Knife 2, KD611L; Olympus, Tokyo, Japan). Finally, the lesion was resected en bloc. ESD was performed using GIF-260J (Olympus, Tokyo, Japan) or GIF-H290 (Olympus, Tokyo, Japan), with the tip attached to a hood (D-201-11804; Olympus, Tokyo, Japan or Elastic Touch, F-30; Top, Tokyo, Japan). Endoscopic closure of ESD-treated site was not performed in all cases. After the first day of ESD, all patients were intravenously administered proton pump inhibitors or potassium-competitive acid blockers. Second-look esophagogastroduodenoscopy (EGD) was performed if the doctor who performed ESD deemed it necessary. In cases requiring a second-look EGD, if bleeding blood vessels or stumps of exposed blood vessels were visually recognized, patients were treated with coagulation and hemostasis. Patients who underwent antithrombotic treatment were managed as per JGES guidelines [24,25,26]. 

### 2.4. Predictive Variables 

Referring to the BEST-J score, PNI, previous reports, and clinical experience, the twenty-one predictors selected were: age, sex, body mass index (BMI), Charlson Comorbidity Index [27], treatment with artificial dialysis [7], use of low-dose aspirin [6,7], use of P2Y12 receptor antagonist (P2Y12RA) [7], use of phosphodiesterase 3 inhibitor (PDE3 inhibitor) [7], use of warfarin [6,7], use of direct oral anticoagulant (DOAC) [7], tumor size > 30 mm [4,7], location of a lesion at lower one-third of the stomach [3,7], presence of multiple tumors [7], undifferentiated cancer [7], tumor pathologically diagnosed as beyond SM1 invasion [7], ESD duration > 120 min [3], failure of en bloc resection, the presence of gastric mucosal atrophy [7], C-reactive protein (CRP) changes before and after ESD, white blood cell (WBC) changes before and after ESD, and PNI [13]. Regarding antithrombotic agents, withdrawal, continuation, and replacement with heparin or low-dose aspirin were considered variables. Mucosal atrophy was evaluated based on the classification of Kimura and Takemoto [28]. PNI was calculated as follows [13]:

*PNI* = 10 *× Alb [g/dL]* + 0.005 × *total lymphocyte count* (1)

### 2.5. Outcome Criteria 

Post-ESD bleeding was the primary observational goal of the study. Based on previous reports [7,29,30], post-ESD bleeding was defined as EGD performance based on findings suggestive of bleeding within 4 weeks of ESD. Findings suggestive of bleeding included the appearance of melena or hematemesis, with a decrease in the hemoglobin level by >2 g/dL. Preventive hemostasis during second-look EGD was not considered for post-ESD bleeding.

### 2.6. Statistical Analysis 

In the model cohort, univariate analysis was performed using Fisher’s exact test or the Mann–Whitney U test. Variables with a *p*-value < 0.05 were eligible for multivariate analysis. The multivariate model incorporated these variables and was evaluated using the backward elimination method by multivariable logistic regression. Variables with a *p*-value < 0.05 were elements of the final model. To avoid multicollinearity, a variance inflation factor (VIF) was used. Variables with VIF > 10 were ruled out. The Hosmer–Lemeshow test was used to measure the fitness of the model. We assigned each variable a score based on the multivariate analysis’s β regression coefficient. The area under the receiver operating characteristic curve (ROC-AUC) was used as a measure of discrimination. To investigate the possibility of overfitting the model, 5-fold cross-validation was executed. The ROC-AUC between the naive prediction model and the validated model was compared for cross-validation. Next, based on the created model, we classified cases in the validation cohort into three groups: low risk, intermediate risk and high risk. We evaluated trends in bleeding rates among them using Cochran–Armitage test. Then, we compared the performance between the created model (now called “modified BEST-J score”) and the BEST-J score by ROC-AUC using DeLong test. All statistical analyses were executed with EZR software [31].

## 3. Results

### 3.1. Patients in the Model Cohort 

In total, 449 patients treated with gastric ESD from January 2011 to December 2021 were enrolled. We divided them into groups with a 2:1 ratio. We assigned 299 patients to the model cohort and 150 to the validation cohort. The clinical characteristics and background of patients in the model cohort are presented in Table 1. The mean ± standard deviation (SD) age was 73 ± 9.04 years and 94 patients were female. The mean ± SD PNI was 49.6 ± 5.19. 

### 3.2. Post-ESD Bleeding in the Model Cohort 

Of the 299 patients in the model cohort, 25 experienced post-ESD bleeding. To detect predictive factors, the post-ESD bleeding and control groups were contrasted (Table 2). The factors include undergoing dialysis (*p* = 0.0020), taking P2Y12RA (*p* < 0.001), tumor size > 30 mm (*p* = 0.0043), lesion location in the lower one-third of the stomach (*p* = 0.0044), and PNI (mean ± SD of post-ESD bleeding vs. control, 46.2 ± 4.79 vs. 50 ± 5.12, *p* < 0.001). Using ROC analysis, the cutoff value of PNI was defined as 47.9. 

According to the multivariable logistic regression analysis, P2Y12RA (odds ratio (OR), 10.5; β regression coefficient (β), 2.35; 95% confidence interval (CI), 2.73–40.3; *p* < 0.001), tumor size > 30 mm (OR, 6.79; β, 1.91; 95% CI, 2.07–22.2; *p* = 0.0016), a lesion in the lower one-third of the stomach (OR, 3.49; β, 1.25; 95% CI, 1.24–9.83; *p* = 0.018), and PNI ≤ 47.9 (OR, 7.48; β, 2.01; 95% CI, 2.34–23.4; *p* < 0.001) (Table 3) had VIF < 10. Undergoing dialysis had no significant difference. The Hosmer–Lemeshow test showed a good fit for the model (*p* > 0.05).

**Figure 2:**
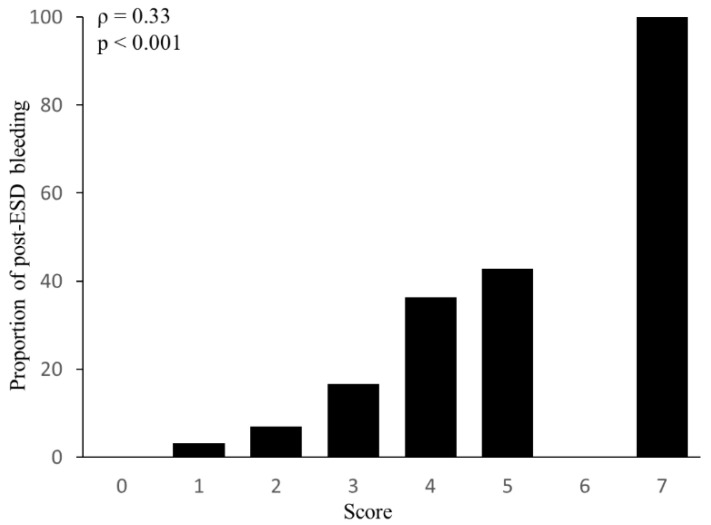
Modified BEST-J score points and percentage of post-ESD bleeding patients at each point in model cohort. Spearman’s rank correlation test was used to determine the correlation coefficient and *p*-value.

Based on the β regression coefficient of multivariable logistic regression, we assigned two points to P2Y12RA, tumor size > 30 mm, and PNI ≤ 47.9 and one point to a lesion located in the lower one-third of the stomach (defined as the modified BEST-J score). Among the patients who had scores of 0, 1, 2, 3, 4, 5, and 7, the proportion of post-ESD bleeding was 0%, 3.23%, 7.02%, 16.7%, 36.4%, 42.3%, and 100%, respectively. Calculated by Spearman’s rank correlation test, the total score was positively correlated with the rate of post-ESD bleeding (rs = 0.33, *p* < 0.001, Figure 2). 

The ROC analysis showed that the sensitivity, specificity, and positive likelihood ratio were 73.9%, 78.1%, and 3.37, respectively. A five-fold cross-validation was performed and ROC-AUC was 0.82 (range, 0.61–0.96) for naïve prediction and 0.71 (range, 0.51–0.90) after cross-validation. 

### 3.3. Comparison between the Modified BEST-J and BEST-J Scores in the Validation Cohort

**Figure 3:**
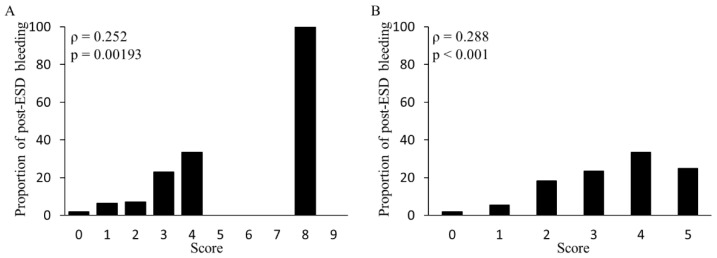
(**A**) BEST-J score points and percentage of post-ESD bleeding patients at each point in the validation cohort. Spearman’s rank correlation test was used to determine the correlation coefficient and *p*-value. (**B**) Modified BEST-J score points and percentage of post-ESD bleeding patients at each point in the validation cohort. Spearman’s rank correlation test was used to determine the correlation coefficient and *p*-value. No patients scored 6 or 7.

The clinical characteristics and background of patients in the validation cohort are presented in Table 1. Of the 150 patients in the validation cohort, 13 experienced post-ESD bleeding. Each patient was scored based on the BEST-J and modified BEST-J scores. The proportion of post-ESD bleeding in patients who scored 0, 1, 2, 3, 4, 5, 6, 7, 8, and 9 according to the BEST-J score was 1.86%, 6.52%, 7.14%, 23.1%, 33.3%, 0%, 0%, 0%, 100%, and 0%, respectively, whereas in patients who scored 0, 1, 2, 3, 4, and 5 according to the modified BEST-J score was 2%, 5.56%, 18.2%, 23.5%, 33.3%, and 25%, respectively (Figure 3).

**Figure 4:**
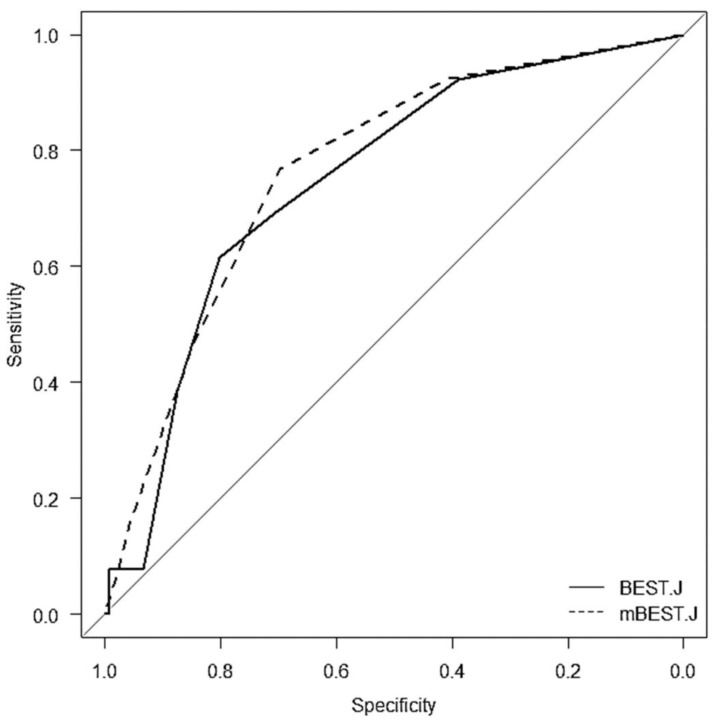
Performance comparison between BEST-J score and modified BEST-J score by receiver operating characteristics curve (ROC) analysis. The sensitivity, specificity, positive likelihood ratio, and area under the receiver operating characteristic curve (ROC-AUC) in BEST-J score were 61.5%, 80.1%, 3.09, and 0.75, respectively. Meanwhile, the sensitivity, specificity, positive likelihood ratio, and ROC-AUC in modified BEST-J score were 69.7%, 76.9%, 3.02, and 0.77, respectively. Regarding ROC-AUC, no significant difference was observed between the BEST-J and modified BEST-J scores (*p* = 0.81).

A positive correlation between post-ESD bleeding rate and the total score was observed in the BEST-J and modified BEST-J scores (rs = 0.25 vs. 0.29, *p* = 0.002 vs. *p* < 0.001); the Hosmer–Lemeshow test showed a good model fit (both *p* > 0.05). Based on modified BEST-J scores, classifying cases with a score of 0,1 as low risk, those with a score of 2 as intermediate risk, those with a scores of 3 or more as high risk, post-ESD bleeding rate tended to increase from low to high risk (*p* < 0.001). Similar trends were observed among low, intermediate, high, and very high groups classified by BEST-J scores (*p* < 0.01). ROC analysis revealed that the sensitivity, specificity, and positive likelihood ratio were 61.5%, 80.1%, 3.09, and 0.747, respectively, for the BEST-J score and 69.7%, 76.9%, 3.02, and 0.767, respectively, for the modified BEST-J score. ROC-AUC was superior to those using the modified BEST-J score; however, no significant difference was observed (0.77 vs. 0.75, *p* = 0.81, Figure 4).

## 4. Discussion

This study showed that the modified BEST-J score could predict post-ESD bleeding based on four variables: the use of P2Y12RA, tumor size > 30 mm, location of a lesion at the lower one-third of the stomach, and PNI ≤ 47.9; the modified BEST-J score performed as well as the BEST-J score in the validation cohort. Post-ESD bleeding is a major complication of ESD. Reportedly, 1.3–11.9% of patients who undergo ESD experience post-ESD bleeding [32], which can occur after the patient is discharged [32], making rapid treatment difficult; hence, predicting post-ESD bleeding is necessary. This study constructed a predictive model for post-ESD bleeding with four variables, including PNI. We showed that it was as accurate as the BEST-J score using a validation cohort. Choe et al. developed a prediction model with three variables: continued use of antithrombotic agents, specimen size ≥ 49 mm, and age < 62 years [17]; however, internal validity was not evaluated. The BEST-J score showed good discrimination (c-statistic was 0.70) and good calibration in the external validation cohort [7]. This model is useful in predicting post-ESD bleeding; however, it should consider 10 factors. The modified BEST-J score can predict post-ESD bleeding with fewer variables; hence, it can predict bleeding more quickly. 

This study showed that only P2Y12RA intake was related to post-ESD bleeding and not the discontinuation of antithrombotic agents. Hakoda et al. reported that P2Y12RA had the highest bleeding risk among antiplatelet agents and continued or discontinued aspirin or PDE3 inhibitor administration did not affect post-ESD bleeding [33]. It has been reported that endoscopic treatment with continued aspirin does not increase the risk of postoperative bleeding [34,35]. PDE3 inhibitors, such as cilostazol, inhibit platelet aggregation without prolonging bleeding time in vivo [36] and do not cause an increased risk of serious bleeding events [37]. Anticoagulants are reported as a risk factor for post-ESD bleeding [6,7]. On the other hand, appropriate adjustment of PT-INR can reduce bleeding risk and anticoagulants are not a bleeding risk when adjusted appropriately for patient background [38,39]. Moreover, several studies show a relationship between gastroprotective agents and anticoagulants. Concomitant use of gastroprotective agents and anticoagulants reduces the risk of gastrointestinal bleeding [40,41]. In this study, all patients who underwent ESD were administered proton pump inhibitors or potassium-competitive acid blockers; hence, these agents may have reduced the risk of post-ESD bleeding caused by anticoagulants. Adenosine-5′-diphosphate promotes wound healing by activating the P2Y12 receptor [42]; hence, P2Y12RA may inhibit wound healing rather than platelet aggregation, which promotes post-ESD bleeding.

Several reports have indicated that preoperative malnutrition is associated with postoperative complications [14,43]. Lee et al. reported that malnourished patients had more wound complications [43]. Among patients 85 years and older who underwent gastric ESD, those with a lower PNI had a poorer survival rate [44]. However, this study is the first to report the association between PNI and ESD complications, including post-ESD bleeding. According to the BEST-J score, chronic renal failure requiring dialysis is considered a high-risk factor for post-ESD bleeding [7]. Severe chronic renal failure with decreased glomerular filtration rate was more common in patients with low PNI [45]; hence, PNI may have also been involved in the BEST-J score. Albumin and lymphocytes contribute to wound healing [46,47], and a low PNI, which reflects these conditions, may have resulted in delayed recovery and bleeding. Preoperative nutritional therapy reduces postoperative complications [48]; hence, nutritional therapy before ESD can reduce postoperative bleeding in patients with a low PNI.

There is an association between tumor size and postoperative bleeding [4,7,17]. Although the definition of tumor size varies among reports, it is defined as at least 30 mm. Hence, this study defined tumor size as ≥ 30 mm. Tumor location is associated with postoperative bleeding; however, studies differ in whether the risk factor is in the upper or lower stomach [4,7]. Chung et al. reported that lesions located in the upper part of the stomach were a risk factor for post-ESD bleeding [4]. However, Hatta et al. reported lesions located in the lower third part of the stomach were a risk factor [7]. This study showed that lesions in the lower part of the stomach were risk factors for post-ESD bleeding. The lower stomach can be exposed to alkaline reflux from the duodenum, causing local gastric acid hypersecretion and resulting damage; therefore, it may be a risk factor for post-ESD bleeding [49].

Including a multicenter study, several studies have been conducted on the timing of bleeding. Nam et al. reported that age, resection size, procedure time, location of lesion in the stomach, erosion, and clopidogrel affected the occurrence of early post-ESD bleeding [50]. In a multicenter study, taking anticoagulants, undergoing dialysis, taking antiplatelet agents, tumor size, multiple tumor, and location of the lesion in the stomach were reported as a risk factor for early post-ESD bleeding [51]. This study showed the use of P2Y12RA, tumor size > 30 mm, and location of a lesion at the lower one-third of the stomach were risk factors for post-ESD bleeding; however, it did not examine whether bleeding occurred early after ESD. Since different risk factors for early post-ESD bleeding were reported by each study, a prospective study with and without risk factors needs to be conducted.

Several limitations are included in this study. First, this was a single-institution retrospective study; therefore, it had a smaller sample size and was more susceptible to selection bias than a multicenter study. In addition, since this study was validated in an internal cohort, it could be subject to selection bias. Moreover, logistic regression analysis requires 10 outcomes for one independent variable; however, the number of post-ESD bleeding events was insufficient for the number of independent variables in the model cohort. There may be a risk of overfitting; therefore, cross-validation and validation with alternative internal cohorts were used to ensure that the modified BEST-J score had sufficient accuracy. Finally, there is a difference in the proportion of cases with variables between model cohort and validation cohort, which may affect the results.

## 5. Conclusions

This study showed that the modified BEST-J score could predict post-ESD bleeding with fewer variables than the BEST-J score. Post-ESD bleeding can be predicted quickly using the modified BEST-J score.

## Figures and Tables

**Table 1:** Clinical features and characteristics in model cohort and validation cohort.

	Model Cohort	Validation Cohort	*p*-Value ^�^
Total	299	150	
Age, mean (SD)	73 (9.04)	74.1 (9.0)	0.197
Males/females, n	205/94	112/38	0.226
BMI, mean (SD)	23.4 (3.38)	23.3 (3.13)	0.883
Charson Comorbidity Index, mean (SD)	1.27 (1.29)	1.44 (1.23)	0.186
Undergoing dialysis, n (%)	4 (1.34)	3 (2.0)	0.691
Antithrombotic agent use, n (%)			
LDA	35 (11.7)	12 (8.0)	0.255
P2Y12RA	15 (5.01)	4 (2.67)	0.323
PDE3 inhibitor	9 (3.01)	2 (1.33)	0.349
warfarin	10 (3.34)	11 (7.33)	0.094
DOAC	8 (2.68)	20 (13.3)	<0.001
Discontinuation of antithrombotic agent	26 (8.7)	26 (17.3)	0.0117
Continuation of antithrombotic agent	5 (1.67)	15 (10.0)	<0.001
Replacement with LDA or heparin	32 (10.7)	6 (4.0)	0.20
Lesion, n (%)			
Tumor size > 30 mm	29 (9.7)	11 (7.33)	0.595
Location at lower 1/3	134 (44.5)	58 (38.7)	0.159
Multiple tumor	24 (8.03)	11 (7.33)	0.854
Undifferentiated tumor	3 (1.00)	51(0.67)	1
Pathologically beyond SM1 invasion	28 (9.36)	5 (3.33)	0.021
ESD procedure, n (%)			
Cutting time > 120 minutes	35 (11.7)	33 (22)	0.00529
Failure of en-bloc dissection	9 (3.01)	4 (2.67)	0.758
Presence of mucosal atrophy	295 (98.7)	138 (92.0)	0.00159
Change before and after ESD, mean (SD)			
Change in CRP (mg/dL), mean (SD)	0.42 (1.05)	0.35 (0.62)	0.451
Change in WBC (/μL), mean (SD)	3290 (2220)	3550 (2250)	0.289
PNI before ESD, mean (SD)	49.6 (5.19)	50.3 (5.25)	0.206

SD; standard deviation, BMI; Body mass index, LDA; Low dose aspirin, P2Y12RA; P2Y12 receptor antagonist, DOAC; Direct oral anticoagulant, SM; Submucosa, ESD; Endoscopic submucosal dissection, CRP; C-reactive protein, WBC; White blood cell, PNI; Prognostic nutritional index. � *p* values were determined using Fisher’s exact test or Mann-Whitny U test.

**Table 2:** Comparison between patients with post-ESD bleeding and without.

	Post-ESD Bleeding	Control	*p*-Value ^�^
	(n = 25)	(n = 274)	
Age, mean ± SD	73 ± 9.05	73 ± 9.17	0.902
Males/females, n	16/9	189/85	0.654
BMI, mean ± SD	22.5 ± 2.57	23.5 ± 3.43	0.28
Charson Comorbidity Index, mean ± SD	1.52 ± 1.9	1.25 ± 1.22	0.214
Undergoing dialysis, n	3	1	0.00197
Antithrombotic agent use, n			
LDA	5	30	0.191
P2Y12RA	6	9	<0.001
PDE3 inhibitor	1	8	0.549
warfarin	1	9	0.588
DOAC	0	8	1
Discontinuation of antithrombotic agent	5	21	0.0527
Continuation of antithrombotic agent	1	4	0.411
Replacement with LDA or heparin	5	27	0.165
Lesion, n			
Tumor size > 30 mm	7	21	0.00439
Location at lower 1/3	17	117	0.0199
Multiple tumor	3	21	0.436
Undifferentiated tumor	0	3	1
Pathologically beyond SM1 invasion	3	25	0.716
ESD procedure, n			
Cutting time > 120 minutes	6	29	0.0945
Failure of en-bloc dissection	1	8	0.549
Presence of mucosal atrophy	25	270	1
Change before and after ESD, mean (SD)			
Change in CRP (mg/dL), mean (SD)	0.661 ± 1.53	0.393 ± 0.998	0.995
Change in WBC (/μL), mean (SD)	3826 ± 2870	3262 ± 2155	0.495
PNI before ESD, mean ± SD	46.2 ± 4.79	50 ± 5.12	<0.001
PNI ≤ 47.9 ^‡^, n	18	92	<0.001

SD; standard deviation, BMI; Body mass index, LDA; Low dose aspirin, P2Y12RA; P2Y12 receptor antagonist, DOAC; Direct oral anticoagulant, SM; Submucosa, ESD; Endoscopic submucosal dissection, CRP; C-reactive protein, WBC; White blood cell, PNI; Prognostic nutritional index. � *p* values were determined using Fisher’s exact test or Mann-Whitny U test. ‡ Cut-off value of PNI were based on receiver operating characteristic analysis.

**Table 3:** Independent risk factors for post-ESD bleeding.^�^.

Predictor	OR (95% CI)	VIF	*p* Value	β Regression Coefficient	Score ^‡^
P2Y12RA use	10.5 (2.73–40.3)	1.05	<0.001	2.35	2
Tumor size > 30 mm	6.79 (2.07–22.2)	1.07	0.00157	1.91	2
Location at lower 1/3	3.49 (1.24–9.83)	1.06	0.018	1.25	1
PNI ≤ 47.9	7.48 (2.39–23.4)	1.07	<0.001	2.01	2

OR; Odds ratio, CI; confidence interval, VIF; Variance inflation factor, P2Y12RA; P2Y12 receptor antagonist. � OR, 95% CI and *p* values were determined using multivariable logistic regression analysis. ‡ Scores were assigned by rounding off the β regression coefficient.

## Data Availability

All data generated or analyzed during the present study are included in this article.

## Abbreviations

ESD: endoscopic submucosal dissection; PNI, prognostic nutritional index; JGES, Japan Gastroenterological Endoscopy Society; BMI, body mass index; P2Y12RA, P2Y12 receptor antagonist; PDE3, phosphodiesterase 3; DOAC, direct oral anticoagulant; SM, submucosal layer; CRP, C-reactive protein; WBC, white blood cell; VIF, variance inflation factor; ROC-AUC, area under the receiver operating characteristic curve; SD, standard deviation; PT-INR, prothrombin time-international normalized ratio.
